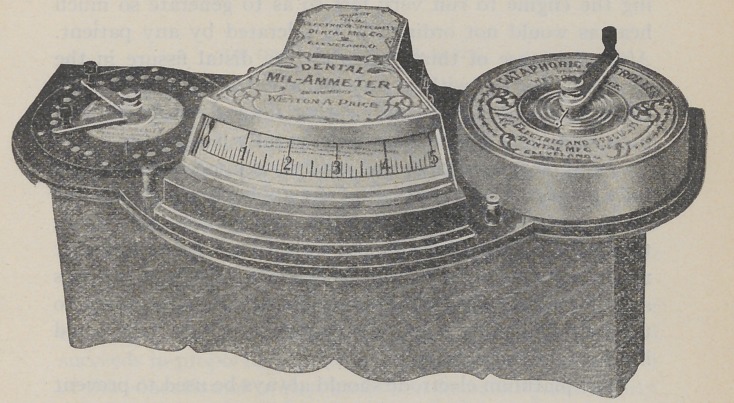# Obtunding Sensitive Dentin with Cataphoresis

**Published:** 1903-02-15

**Authors:** Weston A. Price


### OBTUNDING SENSITIVE DENTIN WITH CATAPHORESIS.

BY WESTON A. PRICE, D.D.S.

The following description of a clinic given before the Ohio State Dental Society at the last meeting, is here given to show that with a properly constituted appliance it is not only possible, but certain and practicable to obtund the most sensitive dentine in a reasonable time, so that cavity preparation can be made as thorough as desired without the least discomfort to the patient.

The patient selected for this operation was a senior dental student of the Ohio Medical University at Columbus. His teeth were so sensitive that he had never been able to have them properly filled. Those filled had been excavated as little as possible and always with so much pain, that he hadn't much faith that he could endure the operation with cataphoresis. Many of the teeth had large cavities and all were so sensitive that he would tolerate no preliminary work with excavators, and even objected to the passing of a ligature between them.

The case selected was a mesial cavity in the first upper molar. It was so sensitive that an effort to remove the loose debris with an excavator caused the patient great pain, as was shown by his effort to slide out of the chair and away from the operator, at the same time the perspiration stood out in great drops all over his forehead. As the tooth adjoining was also decayed, it became necessary to separate them so that the rubber dam could be properly adjusted. The patient would not tollerate the use of a separating file, so a thin spatula was passed through making sufficient separation to permit the dam to be forced through with a ligature. This was considered an ideal case for cataphoresis and one which could in all probability not be handled successfully by any other means of obtunding. It was not possible because of the extent of the decay in the two teeth to make a perfect insulation with the dam alone, as was necessary, and the case was dried as perfectly as was practicable and softened gutta percha packed into the cavity and between the teeth so as to insure insulation at the gum margins. The gutta percha was crowded away from the pulpal wall of the cavity to be treated so as to expose as much of this area of dentine as possible; this, however, left the cavity margins, at the cervical border especially, completely covered by the gutta percha. The application was then made to this area of exposed denture.

The anesthetic used was a saturated solution of Hydrochlorate of Cocain. which was placed in the cavity on a small coil of platinum wire which served as the electrode. The appliance used for producing the cataphoresis was an apparatus which consists of three distinct instruments all combined together in such a manner as to make it possible to have absolute control of the amount of current used and to instantly detect any variation by the eye. The apparatus consists of a battery of thirty dry cells so connected with the *Cell Selector* that any one, or any number of cells, can be called into use as needed. The second part is a new *Controller* which is a complete departure from any now in use which permits one to apply the current so gradually that it is not possible to produce pain by the current from shock.

The third element of the apparatus is the *Mil-Ammeter* which has a scale which can be read in hundred-thousandths of amperes at a distance of thirty feet. The two scales of it read from zero to five mil-amperes. The value of the appliance and its claim to superiority are due to the fact that reliable current is supplied under absolute control, and any slightest variation can be immediately detected by the operator.

In this case the contacts were made and the current applied beginning at zero. The first sensation of any kind which was a slight warmth was shown to be when three one-hundred-thousandths of an ampere had been reached. The voltage was gradually increased by adding one cell at a time, being careful to turn in all the resistance each time a cell was added, and gradually turn it out before the next one was added. In sixteen minutes the tooth tolerated without the slightest pain, forty one-hundred-thousandths of an ampere or four-tenths of a mil-ampere, which is the limit to be used except where the pulp is to be extracted. This is a little more than thirteen times the current which produced the first heat or pain sensation. When this point was reached the current was withdrawn and a disinterested dentist was asked to prepare the cavity for filling. This was done without the slightest complaint from the patient, except when the cervical margin was reached. This portion of the dentine, it will be remembered, was completely covered by the gutta percha insulation. A second application of the current was made for three minutes, and the cutting was resumed and completed without further complaint. To make the test complete the crown fissure was cut out allowing the engine to run very fast so as to generate so much heat as would not ordinarily be tolerated by any patient. After the lapse of thirty minutes, the distal fissure in the tooth was cut out without any inconvenience to the patient, showing the profound and lasting impression which had been made. It does not however, usually last so long.

After the operation the patient expressed himself as fully satisfied with the result of the application. There was never any pain from the time the current was turned on; there was a slight sensation of warmth all the time but it never approached a painful sensation. The patient was so well pleased that he said he would never again submit to the pain of having a tooth prepared for filling if he could have cataphoresis used.

The platinum electrode should always be used to prevent possible electrolysis of the metals and the promotion of other salts which would interfere with the cataphoric distribution of the cocain salt. It is essential to perfect success that absolute insulation be accomplished to prevent dissipation or diversion of the current.

In reply to the inquiry as to whether the dentine would be more quickly obtunded if its entire surface were exposed rather than a small area. I would say that it depends on whether the anesthesia is to be produced from the pulp or only the dentine. If the pulp is anesthetized of course the whole of the dentine will be affected. This is the most reliable method of obtunding the dentine, as the effect is more lasting as well as more profound. If the dentine only is affected it must be apparent that only the portion of the pulp lying immediately beneath the portion of dentine acted upon will be affected, and not the whole organ. In such a case the effects will quickly pass away. It is hoped that with so reliable an apparatus cataphoresis may again come into favor.

## Figures and Tables

**Figure f1:**